# Secretoglobin 1A1 and 1A1A Differentially Regulate Neutrophil Reactive Oxygen Species Production, Phagocytosis and Extracellular Trap Formation

**DOI:** 10.1371/journal.pone.0096217

**Published:** 2014-04-28

**Authors:** Olivier Côté, Mary Ellen Clark, Laurent Viel, Geneviève Labbé, Stephen Y. K. Seah, Meraj A. Khan, David N. Douda, Nades Palaniyar, Dorothee Bienzle

**Affiliations:** 1 Department of Pathobiology, University of Guelph, Guelph, Ontario, Canada; 2 Department of Clinical Studies, University of Guelph, Guelph, Ontario, Canada; 3 Department of Molecular and Cellular Biology, University of Guelph, Guelph, Ontario, Canada; 4 Department of Laboratory Medicine and Pathobiology, University of Toronto, Toronto, Ontario, Canada; 5 Institute of Medical Sciences, University of Toronto, Toronto, Ontario, Canada; 6 Program in Physiology and Experimental Medicine, Lung Innate Immunity Research Laboratory, Hospital for Sick Children, Toronto, Ontario, Canada; University of Tübingen, Germany

## Abstract

Secretoglobin family 1A member 1 (SCGB 1A1) is a small protein mainly secreted by mucosal epithelial cells of the lungs and uterus. SCGB 1A1, also known as club (Clara) cell secretory protein, represents a major constituent of airway surface fluid. The protein has anti-inflammatory properties, and its concentration is reduced in equine recurrent airway obstruction (RAO) and human asthma. RAO is characterized by reversible airway obstruction, bronchoconstriction and neutrophilic inflammation. Direct effects of SCGB 1A1 on neutrophil functions are unknown. We have recently identified that the *SCGB1A1* gene is triplicated in equids and gives rise to two distinct proteins. In this study we produced the endogenously expressed forms of SCGBs (SCGB 1A1 and 1A1A) as recombinant proteins, and analyzed their effects on reactive oxygen species production, phagocytosis, chemotaxis and neutrophil extracellular trap (NET) formation *ex vivo*. We further evaluated whether NETs are present *in vivo* in control and inflamed lungs. Our data show that SCGB 1A1A but not SCGB 1A1 increase neutrophil oxidative burst and phagocytosis; and that both proteins markedly reduce neutrophil chemotaxis. SCGB 1A1A reduced chemotaxis significantly more than SCGB 1A1. NET formation was significantly reduced in a time- and concentration-dependent manner by SCGB 1A1 and 1A1A. SCGB mRNA in bronchial biopsies, and protein concentration in bronchoalveolar lavage fluid, was lower in horses with RAO. NETs were present in bronchoalveolar lavage fluid from horses with exacerbated RAO, but not in fluid from horses with RAO in remission or in challenged healthy horses. These findings indicate that SCGB 1A1 and 1A1A have overlapping and diverging functions. Considering disparities in the relative abundance of SCGB 1A1 and 1A1A in airway secretions of animals with RAO suggests that these functional differences may contribute to the pathogenesis of RAO and other neutrophilic inflammatory lung diseases.

## Introduction

Secretoglobin family 1A member 1 (SCGB 1A1) is a small, secreted protein mainly produced by mucosal epithelial cells in lung and uterus. SCGB 1A1, also known as club (Clara) cell secretory protein (CCSP), was suggested as the standardized nomenclature to define this member of an emerging superfamily of 10 kDa proteins [1]. The SCGB family of proteins exist as disulfide-dependent homodimers that are oriented in an anti-parallel superposition [2]. Structurally, this association generates an internal hydrophobic pocket for binding of lipophilic molecules [3]–[6]._ENREF_5 SCGB 1A1 dimers sequester calcium and phosphatidylcholine, a cofactor and a substrate required for phospholipase A2 activity, respectively [7], [8]. Existence of minor hydrophobic cavities on each side has also been reported, but their functions are unknown. SCGB 1A1 is highly resistant to protease degradation, and stable at high temperatures and pH extremes [9].

The exact roles of SCGB 1A1 in lung physiology and homeostasis are uncertain. Although SCGB 1A1 is not essential for normal lung function, its absence or reduced expression is associated with exacerbation of several inflammatory conditions [10], [11]. Development of synthetic antiflammins based on fusion of conserved structures of SCGB 1A1 and lipocortin-1 yielded compounds with *in vivo* anti-inflammatory properties [12]. Recombinant SCGB 1A1 has been suggested as a therapeutic agent for treating inflammatory diseases [9], and intranasal administration of the protein improved the hospital discharge rate and dependence on supplemental oxygen in premature infants with respiratory distress syndrome [13]. Nevertheless, whether SCGB 1A1 directly affects the function of inflammatory cells is not clearly established [14].

Recurrent airway obstruction (RAO) is an inflammatory airway disease induced by repeated exposure of susceptible horses to inhaled environmental triggers [15]. Influx of neutrophils into the airways is a hallmark of the condition [16]. Horses with RAO have low levels of *SCGB1A1* mRNA in the lungs and low protein concentration in bronchoalveolar lavage (BAL) fluid [17]. The recent discovery that the *SCGB1A1* gene is triplicated in the equine genome, and that the copies evolved differently over time, suggested that different gene products may play important roles in natural adaptation, biological advantage, and possible functional divergence in health and disease [18]. Three-dimensional modeling of SCGBs suggests that the proteins might have acquired different molecular-binding partners [19]

Neutrophils are powerful innate immune cells that rapidly extravasate into injured tissues in response to inflammatory signals. Typically, increased IL-8 in the injured or infected tissue recruit neutrophils via chemotaxis [20]. At the site, activated neutrophils release reactive oxygen species (ROS) or internalize targets by phagocytosis [21]. ROS are also released into the phagocytic vacuoles of neutrophils to kill internalized microbial pathogens [22]. Recent studies show that these innate immune cells also release nuclear components in the form of neutrophil extracellular traps (NETs) to ensnare microbial pathogens [23]. Although the pathways that regulate NET formation, or NETosis, are not clearly defined, this form of cell death occurs at least via two mechanisms: one is dependent on ROS production and the other is dependent on calcium-ion influx [24]. Although NETs are useful, excess NETosis leads to severe tissue damage [25]. SCGB 1A1 appears to reduce neutrophil migration into the lung by high-affinity receptor antagonism and chemoattractant sequestration [26]. It also down-regulates the release of ROS by neutrophils and suppresses activated phagocytosis [27]. However, whether different forms of SCGB 1A1 differentially regulate neutrophil chemotaxis, phagocytosis or NETosis is unknown. Furthermore, neither *in vivo* formation of NETs in inflammatory lung disease nor the differential expression of SCGBs during the disease is known. Here we tested the hypothesis that equine SCGB 1A1 and 1A1A differentially regulate neutrophil functions. We found that (i) SCGB 1A1A suppressed neutrophil chemotaxis significantly more than SCGB 1A1; (ii) both proteins significantly reduced NETosis in a time and concentration-dependent manner; and (iii) SCGB 1A1A but not SCGB 1A1 significantly enhanced oxidative burst and phagocytic activity. During exacerbated RAO with marked neutrophilic airway inflammation, *SCGB1A1* mRNA expression in bronchial epithelium and total SCGB concentration in BAL fluid were significantly reduced in affected animals, and NETs were present in BAL fluid.

## Materials and Methods

### Animals

All research involving animals was approved by the University of Guelph Animal Care Committee (protocol R10-031) and conducted in compliance with Canadian Council on Animal Care guidelines. RAO and control horses ranged in age from 6 to 18 years and were selected according to previously established criteria [17]. Clinical challenge, pulmonary function testing (PFT), and BAL procedures were performed as described [17]. Briefly, horses were maintained outdoor to limit dust and mould exposure preceding the study. Prior to assessment, animals were transferred to a dust-free indoor environment for 24 hours. Then, healthy control and RAO horses in remission were exposed to dusty hay twice a day until clinical signs of respiratory impairment were detected in animals with RAO, and pulmonary function testing indicated pleural pressure change of >15 cm H_2_O. For BAL, horses were restrained in stocks and sedated with 0.5 mL of Sedivet (Romifidine 10 mg/mL, IV; Boehringer Ingelheim, Burlington, ON). Briefly, a 13-mm diameter, 180-cm long sterile bronchoscope (Olympus, Tokyo, JP) was inserted through a nostril and passed to the level of the carina. A 0.2% lidocaine solution (AstraZeneca, Mississauga, ON) was intermittently applied via the endoscope to reduce coughing and discomfort. The bronchoscope was wedged in a 2^nd^ or 3^rd^ generation bronchus, and 120 mL of warm sterile saline was instilled and re-aspirated. The BAL fluid was filtered, cell concentration was determined with a particle counter (Z2 Coulter counter, Beckman Coulter, Mississauga, ON), and the sample was centrifuged for 10 min at 500 × g to pellet cells. Protein concentration in cell-free supernatant was determined with using a NanoDrop 2000 photometer (Thermo Fischer Scientific, Cooksville, ON).

### Genome data sources

*SCGB1A1* nucleotide sequences were obtained from the NCBI (Bethesda, MD) database as follows; *E. caballus SCGB1A1*, JQ906260.1; *SCGB1A1A*, JQ906261.1. Sequence alignments were generated using Geneious Pro software (Geneious, Auckland, NZ), as previously reported [18].

### Cloning and production of equine recombinant SCGB 1A1 and 1A1A

Total RNA was isolated from equine frozen lung tissues (RNeasy, Qiagen, Mississauga, ON) and reverse transcribed into complementary DNA (cDNA) _ENREF_2[18]. *SCGB1A1* and *SCGB1A1A* coding regions were obtained by PCR amplification using the following primers (Sigma-Aldrich): *SCGB1A1-*F, 5′- GAA ATC TGC CAG AGC TTT GCA GAC ATC ATT CAA GGC C-3′; *SCGB1A1*-R, 5′-GTC ACC TGC AGG CTA AGC ACA CAG TGG GCT CTT TGC-3′; *SCGB1A1A*-F, 5′-GGA ATC TGC CAG AGA TTG GTA GGC ATC GTT CAA GCC C-3′; *SCGB1A1A*-R, 5′-GTC ACC TGC AGG CTA AGC ACA CAG TGG GCT CTC TAC-3′. Primers were designed with XmnI and SbfI restriction sites for cloning into the pMAL-c5X expression vector (New England BioLabs, Mississauga, ON). This vector is designed to produce maltose-binding protein (MBP) fusion proteins without adding vector-derived residues. Amplifications were carried out using a Platinum Taq polymerase PCR kit (Invitrogen, Mississauga, ON). Each reaction was performed in a final volume of 25 µL, including 2 µL of 10 × PCR buffer, 0.2 mM dNTPs, 2 mM MgCl_2_, 0.3 µM of each primer, 2 U of Platinum Taq, and 1 µL of template cDNA (100 ng). Conditions for amplification were 1 min at 94°C followed by 30 cycles of 94°C for 30 sec; 62°C for 30 sec; and 72°C for 90 sec, followed by a final extension at 72°C for 7 min. PCR products were subjected to electrophoresis; bands of appropriate size were excised from the gel, purified (Qiagen) and submitted for automated sequencing (Laboratory Services Division, Guelph, ON). Amplicons were analyzed in duplicate using reverse and forward primer sequencing strategies. *SCGB1A1* and *SCGB1A1A* cDNAs were blunted (New England BioLabs), digested with SbfI, and individually ligated in the XmnI/SbfI site of the pMAL-c5X vector. Both vectors were transformed into NEB Express enhanced competent *Escherichia coli* BL21 (New England BioLabs), incubated overnight at 37°C, and selected on Luria-Bertani agar plates containing 100 µg/mL ampicillin. Ten clones were grown on agar plates overnight for each construct. Plasmids were screened by PCR using primers pMAL-F, 5′-GCG CAG ACT AAT TCG AGC TC-3′ and pMAL-R, 5′-CCT ACT CAG GAG AGC GTT C-3′. PCR products were separated by electrophoresis, excised, purified, and sequenced. Sequences were analyzed with Geneious Pro software (Geneious) to confirm integrity and proper directional insertion of the insert into the vector.

Transformed *E. coli* cells were grown overnight in 10 mL of Rich Medium (yeast/tryptone/NaCl/glucose/ampicillin) at 37°C with gentle shaking. Five milliliters of the culture were transferred to flasks containing 500 mL of Rich Medium and incubated 3 hours with gentle shaking. Isopropyl-beta-D-thiogalactopyranoside (IPTG) was added to a concentration of 3 mM and the culture was incubated at 37°C for 2 hours. Cells were pelleted by centrifugation at 4,000 × g for 20 min at 4°C, resuspended in 25 mL of column buffer (20 mM Tris-HCl pH 7.4, 1 mM EDTA, 200 mM NaCl, 1 mM DTT) and frozen at −20°C overnight. Cells were thawed in an ice-water bath, sonicated on ice, and centrifuged at 20,000 × g for 20 min at 4°C. The supernatant was collected, diluted to 1∶6 with column buffer, passed through an amylose column (New England BioLabs), washed with column buffer, and eluted with 10 mM maltose enriched column buffer. The eluate was then incubated with 1% Factor Xa for 44 hours at room temperature. The protein mix was concentrated using 10K Amicon columns (EMD Millipore, Billerica, MA) and purified using Fast Protein Liquid Chromatography (FPLC). A volume of 500 µL of purified proteins were passed through a HiLoad 26/60 SuperDex 200 column attached to ÄKTA explorer FPLC system (GE Healthcare, Baie d’Urfé, QC), using a filtered FPLC buffer (20 mM Tris-HCl pH 7.4, 150 mM NaCl). Fractions containing SCGB 1A1 or SCGB 1A1A were passed through an amylose column (New England BioLabs), ran on EndoTrap columns (Hyglos, Bernried, Germany) to remove endotoxin, washed with PBS buffer, and stored at −80°C. Recombinant SCGB 1A1 and SCGB 1A1A preparations tested negative for endotoxin using the *Limulus polyphemus amoebocyte* lysate assay (Sigma-Aldrich, Burlington, ON).

### Phagocytosis and oxidative burst assays

Equine blood was collected with acid citrate dextrose (ACD) as anticoagulant and kept at room temperature for less than one hour. Then, 40 mL of blood diluted 1/3 (v/v) into PBS/EDTA was layered on top of 10 mL of Ficoll-Paque PLUS (GE Healthcare). After centrifugation at 500 × g for 30 min, erythrocytes were removed by hypotonic lysis for 45 sec in water. Samples were centrifuged again at 300 × g and the upper layer containing lysed erythrocytes was removed. This step was repeated if the sample still appeared reddish. The resulting white neutrophil pellet was suspended at 5 × 10^6^ cells/mL in PBS/FBS 10% (v/v).

Neutrophils (5 × 10^5^ cells) were incubated in the presence (250, 500, or 1000 ng/mL) or absence of either SCGB 1A1 or SCGB 1A1A (n≥5 horses) in a total volume of 200 µL for 30 min at 37°C with gentle agitation, and centrifuged at 300 × g for 10 min. The supernatant was removed and the pellet was re-suspended in 100 µL of a fresh solution of PBS/FBS 10% (v/v). H_2_DCFDA (Molecular Probes, Eugene, OR, USA) was added to each flow cytometry vial (BD Biosciences, Bedford, MA, USA) at a final concentration of 10 µM and incubated with gentle agitation for 15 min at 37°C. Next, 25 ng/mL of phorbol myristate acetate (PMA, Sigma, St-Louis, MO, USA) was added, and cells were incubated for an additional 15 min. In phagocytosis assays, neutrophils were treated as above. After re-suspension in PBS/FBS 10% (v/v), neutrophils were incubated with 2.7 × 10^7^ fluorescent beads (FluoSpheres sulfate microspheres, 1.0 µm, 505/515, Invitrogen) and 1/5 (v/v) filtered normal horse serum (NHS) to opsonize the beads. Samples were incubated 30 min at 37°C with gentle agitation and cells were analyzed by flow cytometry (FACscan) using Cell Quest software (BD Biosciences). Neutrophils were discriminated by forward and side light scatter.

### Microchemotaxis assay

Freshly isolated neutrophils (n = 5 horses) were loaded with ng/μL of calcein AM (Molecular Probes, Life Technologies, Burlington, ON) during 30 min at 37°C with gentle agitation, centrifuged at 300 × g for 10 min, and re-suspended in a fresh solution of PBS/FBS 10%(v/v). Neutrophils were then pre-treated for 30 min at 37°C with 0, 250, 500, or 1000 ng/mL of SCGB 1A1 or SCGB 1A1A. Twenty μL of sample was plated onto the ChemoTx microplates (Neuro Probe, Maryland, MD). The lower chambers were loaded with 29 µL of a PBS/FBS 10%(v/v) solution supplemented with 0.3 µg/mL of recombinant human IL-8 (R&D Systems, Minneapolis, MN) as chemoattractant. Serial dilutions of the cell suspension (1 × 10^5^, 7.5 × 10^4^, 5 × 10^4^, 2.5 × 10^4^, 1.25 × 10^4^, and 0 cells) were added to the chambers for a calibration curve, and all samples were analyzed in triplicate. Each microplate was incubated at 37°C (5% CO_2_) for 45 min to allow migration. The filter membrane was then rinsed with PBS to remove non-migrating neutrophils, and fluorescence in wells was measured with a Synergy HT microplate reader using Gen5 analysis software (BioTek, Vermont, USA).

### NETosis assays

For NETosis assay, neutrophils were isolated using a modified version of the Polymorphprep protocol (Axis-Shield, Dundee, UK) since EDTA is preferred over ACD anticoagulant for NET assays. Briefly, 20 mL of equine blood was collected into EDTA, layered on an equal volume of Polymorphprep and centrifuged at 500 × g for 35 min at room temperature. Polymorphonuclear cells (PMN) were harvested, mixed with one volume of HEPES-buffered saline (0.42% NaCl, 5 mM HEPES-NaOH, pH 7.4), and centrifuged for 10 min at 400 × g. The pellet was rapidly re-suspended in 10 mL of a 0.2% NaCl solution (max 30 sec), mixed with 10 mL of 4 × HEPES-buffered saline (1.6% NaCl, 20 mM HEPES-NaOH, pH 7.4), and collected by centrifugation. These steps were repeated twice. Cells were washed with 2 × HEPES-buffered saline solution (0.85% NaCl, 10 mM HEPES-NaOH, pH 7.4), harvested by centrifugation and re-suspended in Roswell Park Memorial Institute (RPMI) medium at a concentration of 7 ×10^5^ cells/mL. Each well of a 96-well special optical plate (Corning, Lowell, MA) was loaded with 50 µL of cells and incubated for 30 min at 37°C with 0, 125, 250, 500, 1000, or 2000 ng/mL of SCGB 1A1 or 1A1A in triplicate wells. After incubation, an additional 50 µL of cells supplemented with 5 µM of cell impermeant Sytox Green Nucleic Acid Stain (Invitrogen) was added to each well. In two replicate plates, triplicate wells with cells were treated with either PMA (20 to 120 nM; ROS-dependent) or A23187 ionophore (4 µM; Sigma-Aldrich; ROS-independent) to stimulate NETosis. [24], [28] NET formation and subsequent DNA release was monitored by quantifying fluorescence emission generated by the interaction of Sytox Green stain with extracellular DNA. Total fluorescence was measured using a Gemini EM fluorescence microplate reader (Molecular Devices, Sunnydale, CA). For fluorescence imaging, cells were fixed in 4% (v/v) paraformaldehyde and incubated with Sytox Green DNA stain (Molecular Probes). The cells were washed and permeabilized with PBST (PBS containing 0.05% Tween-20). The following antibodies were used: anti-histone H3 (1∶1000; Abcam, Cambridge, MA), anti-myeloperoxidase (1∶2000; Abcam), anti-SCGB 1A1 (1∶400; [27]), and Alexa Fluor 647 secondary antibodies (1∶10,000; Molecular Probes). Images were captured using a Zeiss Axiovert 200 with a spinning disk confocal scan head (Carl Zeiss, Oakville, ON), equipped with a Hamamatsu C9100-13 EM-CCD camera (Hamamatsu, Middlesex, NJ) and a 40×/0.95 water immersion objective. Volocity software was used for image analysis (PerkinElmer, Cambridge, MA).

### Analysis of *in vivo* NET-derived DNA and NET assays

BAL fluid from six control and seven age-matched RAO-susceptible horses pre- and post-challenge was analyzed by agarose gel electrophoresis, as described [29]. Briefly, each sample was separated on gels with or without proteinase K (PK; 0.2 mg/mL). Presence of nucleic acids in BAL fluids was verified by DNase (50 µg/ml), RNase (250 µg/ml), or dual treatment for one hour at 37°C in the presence of 3 mM CaCl_2_. Samples were subjected to electrophoresis and DNA was visualized using SYBR Safe DNA gel stain (Invitrogen). Images were acquired with a Chemidoc XRS gel imaging system (Bio Rad, Mississauga, ON) and analyzed using Image Lab 4.1 software (Bio-Rad). Western Blot for citrullinated histone H3 (CitH_3_) was performed using the anti-histone H3 antibody (citrulline R2+R8+R17, Abcam).

### Quantitative *SCGB1A1* and *SCGB1A1A* gene expression

A specific cDNA band of 200 bp was amplified for *SCGB1A1* and *SCGB1A1* using the following primers: *SCGB1A1* forward UGrt-2 F (5′-GCT TTG CAG ACA TCA TTC AAG GCC-3′) and reverse UGrt-2R (5′-CTA AGC ACA CAG TGG GCT CTT TG-3′); *SCGB1A1A* forward UGrt-3 F (5′-GAT TKG TAG GCA TCG TTC AAG CCC-3′) and reverse UGrt-3R (5′-CTA AGC ACA CAG TGG GCT CTC TA-3′). A 254 bp equine glyceraldehyde dehydrogenase (*GAPDH*) gene product served as an internal control using forward GAP-F (5′-GTT TGT GAT GGG CGT GAA CC-3′) and reverse GAP-R (5′-TTG GCA GCA CCA GTA GAA GC-3′) primers. The equine *18S* ribosomal RNA gene (203 bp) was used as an additional internal control using forward 18S-F (5′- ATG CGG CGG GGT TAT TCC-3′) and reverse 18S-R (5′-GCT ATC AAT CTG TCA ATC CTG TCC-3′) primers. Quantitative PCR amplifications were performed in a master mix containing 10 µL of SYBR Green 2 × PCR buffer (Qiagen), 8 µL of PCR-grade water, 0.4 µM of each forward and reverse primer, and 1 µL of cDNA template (1 ng/μL). Conditions for amplification were 7 min at 95°C followed by 45 cycles of 95°C for 15 sec; 61°C for 15 sec; 72°C for 20 sec using a LightCycler 480 instrument (Roche, Montreal, QC). *SCGB1A1* primer specificity and identity of the PCR products was confirmed with a melting curve (95°C for 5 sec; 95 to 45°C; 40°C for 10 sec) and sequence analysis, respectively. For each gene, a series of purified cDNA PCR product dilutions (100, 10, 1, 0.1, 0.01, 0.001 ng/μL) was amplified and the average crossing point of each dilution was used to derive a standard curve. *SCGB1A1*, *SCGB1A1A*, *GAPDH*, and *18S* cDNA were amplified in triplicate for each sample along with standard curve calibrators. The cycle threshold (C_T_) was determined and normalized to that of the *GAPDH*, and *18S* housekeeping genes (ΔC_T_). The comparative threshold method (ΔΔC_T_) was used to measure *SCGB* gene expression. Data were analyzed using LightCycler 480 SW 1.5 software (Roche).

### SCGB ELISA

BAL fluid protein concentration was measured (Nanodrop) and diluted with 50 nM carbonate buffer (pH 9.6) to 15 ng/μL. The resulting samples (100 µL) were added into triplicate wells of a NUNC MaxiSorp polystyrene ELISA plate (Thermo Fischer Scientific) and incubated overnight at 4°C. Wells were washed with 0.1% (v/v) Tween-20 in 50 mM PBS, pH 7.4 (T-PBS) several times and blocked with 5% (w/v) powdered skim milk in T-PBS for 6 hours at 4°C. Following three washes with T-PBS, 200 µL of SCGB antibody (diluted 1∶350 in T-PBS/1% (w/v) skim milk powder) was added to each well, and samples were rotated overnight at 4°C on an orbital shaker. After five washes in T-PBS, 100 µL of polyclonal swine anti-rabbit HRP antibody (Dako) diluted 1∶8,000 in T-PBS/1%(w/v) skim milk powder was added to each well. Samples were incubated at room temperature for 30 min and then washed. Finally, 100 µL of 3,3′, 5,5′-tetramethylbenzidine solution (Thermo Fischer Scientific) was added as substrate to each well, the plates were incubated at room temperature for 15 min, and the reaction was terminated by addition of 100 µL of 2 M sulfuric acid. Absorbance was measured at 450 nm. Incubation of samples with pre-immune rabbit serum served as a negative control, and readings from blank wells were used to determine background signal. Serial dilutions of recombinant equine SCGB (0, 1.25, 2.5, 5, 10, 20, 40 and 280 ng/μL) in carbonate buffer were used as standards. All samples and standards were tested in triplicate.

### Statistical analysis

Values were expressed as means ± SEM, and data were analyzed as a 2-factor split-plot in a randomized block design with SCGB treatment as the primary factor. Residual plots were constructed, and formal tests of normality of the residuals were applied (SAS, Cary, NC). Two-way ANOVA with repeated measures, and Bonferroni posttests were performed for NETosis data. Graphs were prepared with Prism5 software (GraphPad, San Diego, CA); *p*≤0.05 was considered significant.

## Results

### Cloning, expression and purification of equine recombinant SCGB 1A1 and SCGB 1A1A

**Figure 1:**
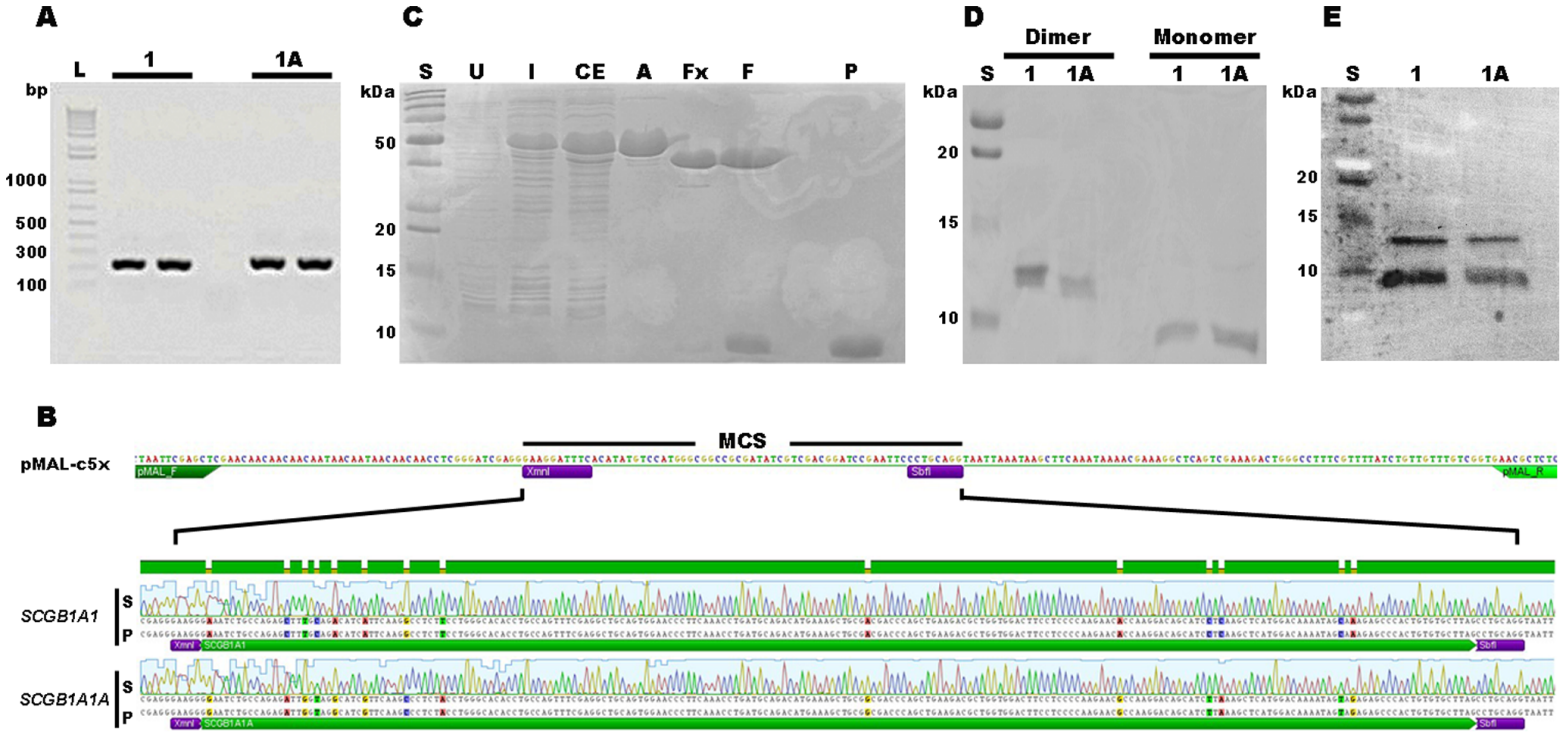
Cloning, expression and purification of equine recombinant SCGB 1A1 and SCGB 1A1A proteins. (A) *SCGB1A1* (“1”) and *SCGB1A1A* (“1A”) partial ORFs were amplified from equine lung cDNA preparations. A unique band of appropriate size (225 bp) was amplified for each gene. L  =  1 Kb+ DNA ladder. (B) Fragments were digested with XmnI and SbfI restriction enzymes (purple boxes) and inserted into the multiple cloning sites (MCS) of the pMAL-c5X expression vector (top). DNA from the transformed colonies was submitted for sequencing to determine the presence, integrity, orientation and suitable translational reading frame of the insert. *SCGB1A1* and *SCGB1A1A* sequenced (S) products showed proper orientation and 100% identity to the predicted (P) sequences. (C) Fractions collected during the purification steps of SCGB 1A1 and SCGB 1A1A were analyzed by SDS-PAGE. A fusion protein was apparent in extracts from IPTG-induced (I) but not un-induced (U) colonies. A crude extract (CE) was collected from induced cells and purified by affinity chromatography, using an amylose (A) column. The eluted fractions were pooled and incubated with Factor Xa protease (Fx) to cleave the fusion proteins. Fx was removed by FPLC (F), and MBP (42.5 kDa) was removed by additional passage on an amylose column from which pure (P) recombinant proteins (7 kDa) were collected. (D) Purified SCGB 1A1 and SCGB 1A1A proteins form dimers that dissociate under reducing and denaturing conditions. (E) Identity of dimers and monomers was confirmed by Western blot analysis. (C, D, E) S  =  Precision plus protein standard (dual color).

To generate recombinant SCGBs, the cDNAs coding for 1A1 and 1A1A mature proteins, excluding the signal peptide sequences, were obtained by PCR amplification from lung biopsies. For each reaction, a single band of expected size (225bp) was detected by gel electrophoresis (Figure 1A), extracted, purified, and inserted into the pMAL-c5X expression vector (Figure 1B). Newly generated *SCGB1A1* and *SCGB1A1A* expression vectors were then transformed into competent cells and at least five independent transformants were sequenced to confirm the identity and proper integration of the insert, as shown in Figure 1B. The sequencing chromatograms revealed 100% identity with *SCGB1A1* and *SCGB1A1A* (NCBI JQ906260.1 and JQ906261.1, respectively). A transformant was selected, inoculated, cultured and harvested for each construct. A crude extract was prepared from individual cultures and subjected to affinity and gel filtration chromatography to retrieve the purified fusion proteins. Approximately 30 to 50 µg of purified protein was obtained per liter of culture. To assess purity, multiple fractions were collected at defined steps and loaded on SDS-PAGE gels. SCGB 1A1 and 1A1A fusion proteins (48 kDa) were detected in extracts induced with isopropylthiogalactoside (IPTG) but not in the un-induced fractions (Figure 1C). Addition of Factor Xa protease to the fusion protein preparations generated two bands of 40 and 7 kDa, indicating proper cleavage of maltose binding protein (MBP) from either SCGB 1A1 or 1A1A. Both recombinant proteins formed homodimers (14 kDa) in non-reducing conditions, and monomers in reducing and denaturing SDS-PAGE (Figure 1D). Results indicated that structure and conformation of the recombinant proteins were conserved, allowing dimerization via the cysteine residues located at both ends of the polypeptide. Western blot analysis under denaturing and mild reducing conditions was performed to confirm the identity of SCGB 1A1 and SCGB 1A1A monomers and dimers (Figure 1E).

### SCGB 1A1 and 1A1A differentially modulate neutrophil oxidative burst and phagocytosis

**Figure 2:**
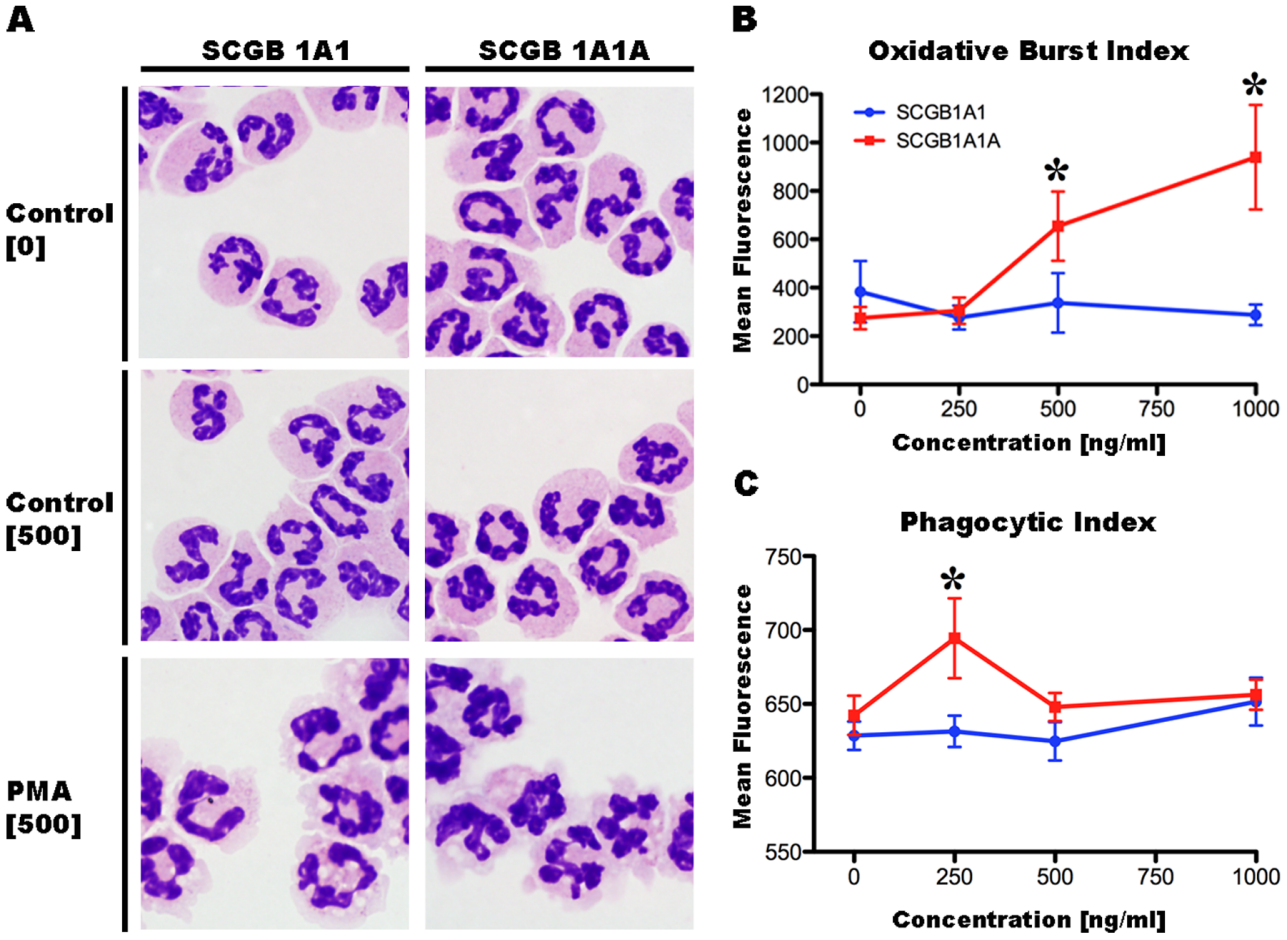
SCGB 1A1 and 1A1A affect neutrophil oxidative burst and phagocytosis. (A) Blood-derived neutrophils were pre-incubated with endotoxin-free SCGB 1A1 or 1A1A prior to oxidative burst and phagocytosis assays. As seen in the two upper panels, control [0, PBS] or SCGB 1A1 and 1A1A [500 ng/mL] treatment did not induce morphological changes in neutrophils, while PMA stimulation triggered neutrophil activation characterized by larger size, membrane ruffles, and formation of cytoplasmic vacuoles (last panel). (B) Increasing concentrations of SCGB 1A1A (red line) stimulated generation of oxidation-dependent neutrophil fluorescence. Increases at 500 and 1000 ng/mL were significantly different from baseline and 250 ng/mL, and relative to SCGB 1A1 (blue line). (C) Both SCGBs had a tendency to increase neutrophil phagocytic activity, but statistically significant differences from each other or baseline were for SCGB 1A1A at 250 ng/mL. Bars  =  SEM. *  =  *p*<0.05

To test the effect of SCGB 1A1 and 1A1A on ROS production and phagocytosis, flow cytometric assays were performed on blood-derived neutrophils freshly isolated from healthy horses. Neutrophil activation and ROS production was initiated with phorbol myristate acetate (PMA), which increased fluorescence intensity >6 fold relative to PBS-treated controls. Cell preparations treated with PMA had characteristic morphological changes of activated neutrophils such as membrane ruffling and cytoplasmic vacuolation (Figure 2A). Addition of either the fluorescent substrate dichlorodihydrofluorescein diacetate (H_2_DCFDA) or SCGB 1A1 or SCGB 1A1A had no effect on neutrophil morphology. A marked and dose-dependent increase in fluorescence was observed in SCGB 1A1A treated neutrophils (Figure 2B), whereas increasing amounts of SCGB 1A1 had minimal effect. SCGB 1A1A-induced pro-oxidant effects differed significantly from those of SCGB 1A1 at 500 (338±123 vs 655±143, *p* = 0.0012) and 1000 ng/mL (288±42 *vs.* 940±216; *p*<0.0001).

The effect of SCGB 1A1 and 1A1A on neutrophil phagocytic activity was analyzed by quantifying fluorescent bead internalization after incubation of neutrophils and beads with or without recombinant proteins. Compared to SCGB 1A1, SCGB 1A1A pre-incubation (250 ng/mL) significantly increased fluorescence (631±11 *vs*. 694±27; *p* = 0.0028; Figure 2C). Significant differences were not detected at 500 and 1000 ng/mL of SCGB 1A1A.

### SCGB 1A1 and 1A1A inhibit IL-8-driven neutrophil chemotaxis

**Figure 3:**
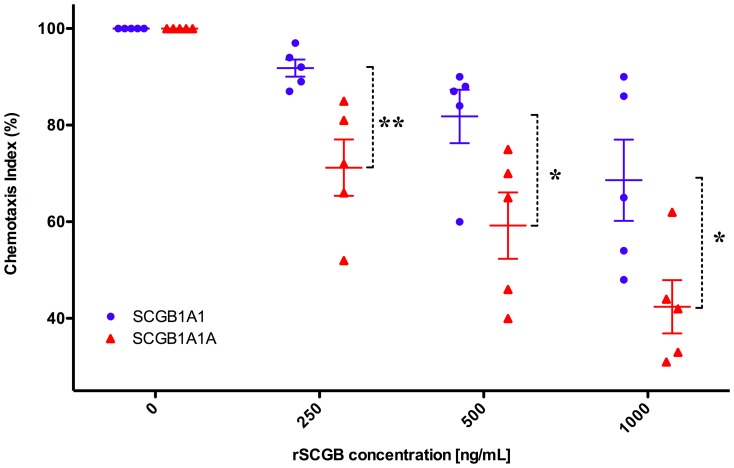
Chemotaxis of equine neutrophils after exposure to recombinant SCGB 1A1 and SCGB 1A1A. Neutrophils were pre-incubated with various concentrations of equine recombinant SCGB 1A1 or 1A1A, and deposited on chemotaxis chambers with IL-8 as chemoattractant. Chemotaxis of untreated neutrophils was assigned 100%. *  =  *p*<0.04, **  =  *p*<0.02, bars  =  SEM; repeated measures ANOVA.

The effect of recombinant SCGB 1A1 and SCGB 1A1A on chemotactic activity of equine neutrophils was characterized using a microchemotaxis plate assay. Cells were pre-treated with serially diluted concentrations of SCGB 1A1 or SCGB 1A1A (0, 250, 500, 1000 ng/mL) and deposited on IL-8 filled chemotaxis chambers. Both recombinant proteins significantly attenuated IL-8 induced migration of neutrophils in a dose-dependent manner (Figure 3), but SCGB 1A1A inhibited chemotaxis significantly more than SCGB 1A1. SCGB effects were most different at 250 ng/mL concentration, where 91.8±4.0% of SCGB 1A1 pre-treated cells had migrated, but only 71.2±13.1% of SCGB 1A1A pre-treated cells showed chemotaxis function (*p*<0.02). At higher concentrations, there was progressively greater inhibition of neutrophil migration, and SCGB 1A1A continued to have a more pronounced effect than SCGB 1A1 (*p*<0.04).

### SCGB 1A1 and 1A1A inhibit NET formation *ex vivo*

**Figure 4:**
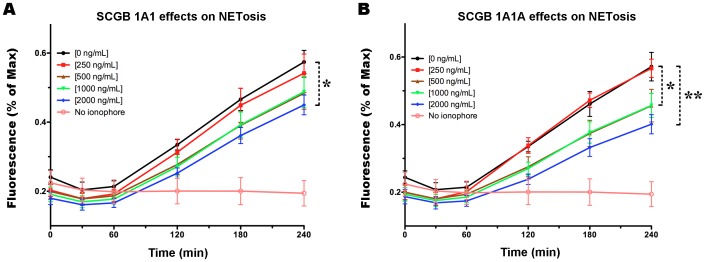
*Ex vivo* NET formation was altered by exposure to SCGB 1A1 (A) or 1A1A (B). Neutrophils were pre-treated with different concentrations of SCGBs and NETosis was induced with calcium ionophore. NET formation was monitored by fluorescence plate reader assay. Bars  =  SEM. *  =  *p* <0.05, **  =  *p* <0.001, repeated measures ANOVA with Bonferroni post tests (0 *vs.* different concentrations of SCGBs). Background neutrophil control values with no ionophore are included in both graphs.

To determine whether SCGBs affect NETosis, we investigated the kinetics of NET formation in neutrophils treated with different concentrations of recombinant SCGBs by determining fluorescence in plate reader assays and confocal microscopy [29]. Plate reader assays detect extracellular DNA because Sytox green is impermeable to live cells and apoptotic cells. Both SCGB 1A1 and 1A1A inhibited calcium ionophore A23187-induced NET formation by equine neutrophils *ex vivo* (Figure 4A and 4B). A dose-dependent (0–2000 ng/mL) and time-dependent (0–4 h) inhibition of NET formation was observed in plate reader assays (n = 7 different samples in triplicate; *p*<0.05). There was a statistically significant interaction between the concentration of SCGB 1A1A and time (*p*<0.05). Higher concentrations of SCGB 1A1 and 1A1A reduced NETosis by approximately 22% and 30%, respectively. Although SCGB 1A1A appeared to inhibit NETosis better than 1A1, differences in the abilities of these two proteins up to 2000 ng/mL were statistically not different. Of note, PMA concentrations of 20 to 160 nM failed to trigger NET formation in neutrophils (data not shown), suggesting alteration or loss of this ROS-dependent NETosis pathway in horses.

**Figure 5:**
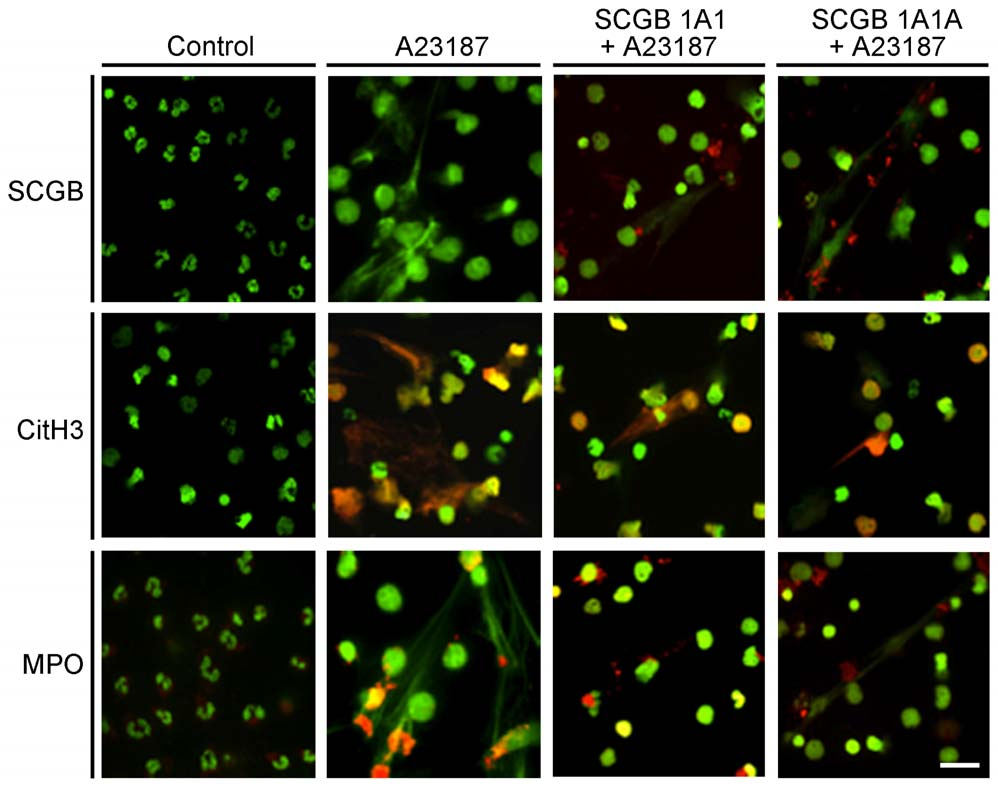
SCGB 1A1 and 1A1A inhibit calcium ionophore (A23187) mediated NETosis in equine neutrophils. Cells obtained from healthy horses were pre-incubated with PBS, SCGB 1A1, or SCGB 1A1A. PBS-treated cells were incubated in the absence (control) or in the presence of calcium ionophore (A23187) to stimulate NETosis. Immunofluorescence analysis revealed discrete nuclei and NETs as string-like structures (green DNA stain). SCGB 1A1 and 1A1A recombinant proteins were detected using SCGB antibody (red, top panel). Neutrophils stimulated with A23187 induced the expression of NETosis markers, including CitH_3_ and MPO (red, middle and bottom panel). Note reduction of NETs in SCGB 1A1 and 1A1A treated cells. Original magnification 40 ×, Bar 20 µm.

At the end of the NETosis assays, neutrophils were fixed in the presence of Sytox Green, washed, permeabilized and immunostained to evaluate the presence of SCGBs and two established NET markers, CitH_3_ and MPO, in the cells and on the NETs. During fixation, Sytox Green enters the cells and also stains intracellular DNA. Ionophore A23187 stimulation yielded strong and diffuse nuclear staining suggestive of chromatin decondensation and NET release, while untreated cells had typical segmented horseshoe-shaped nuclear morphology (Figure 5). SCGB 1A1 and 1A1A staining was detected only in SCGB treated cells, consistent with lack of endogenous expression of SCGBs in neutrophils (Figure 5, top panel, SCGB antibody recognizes an epitope shared by both recombinant proteins). Detection of CitH_3_ staining was restricted to A23187 stimulated cells, consistent with ongoing nuclear DNA externalization and histone deimination (Figure 5, middle panel). MPO staining was detected rarely in neutrophil preparations prior to stimulation, but was prominent on string-like DNA following A23187 treatment (Figure 5, bottom panel). Importantly, there was a substantial reduction in extracellular DNA filaments (green dye) in A23187-stimulated cells pre-treated with SCGB 1A1 and 1A1A relative to positive control cells. Altogether, immunofluorescence analysis showed inhibition of NETosis by SCGB 1A1 and 1A1A proteins.

### *SCGB* mRNA and protein are reduced in animals with RAO

**Figure 6:**
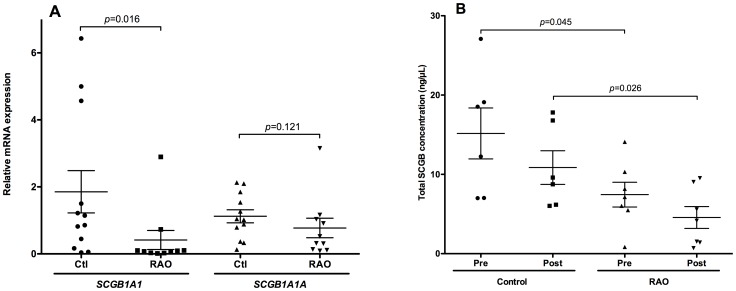
(A) *SCGB1A1* and *SCGB1A1A* mRNA levels were determined by quantitative RT-PCR in equine bronchoscopic biopsies. *SCGB1A1* expression levels were significantly lower (*p* = 0.016) in animals with RAO than in control animals. Results were normalized to that of glyceraldehyde 3-phosphate dehydrogenase (*GAPDH*) mRNA and 18S ribosomal RNA (*RN18S*). Experiments were performed in triplicate and results are presented as mean ± SEM of five (RAO) or six (control) animal per group. (B) Total SCGB concentration as measured by ELISA in BAL fluid was lower in horses with RAO than control horses pre- and post-challenge.

*SCGB1A1* and *SCGB1A1A* transcripts and total SCGB protein concentrations were measured by quantitative real-time PCR of cDNA preparations obtained from bronchial biopsies and by ELISA in BAL fluid, respectively. Previous studies showed that *SCGB1A1* was reduced in horses with RAO compared to those without lung disease, but did not assess expression of individual genes [17]. Here, SCGB expression was compared to glyceraldehyde 3-phosphate dehydrogenase (*GAPDH*) mRNA and 18S ribosomal RNA (*RN18S*) as internal controls. *SCGB1A1* mRNA concentration was significantly lower in animals with RAO compared to controls (*p* = 0.016, Figure 6A), while expression of *SCGB1A1A* was minimally changed. Total SCGB concentration in BAL was significantly lower in horses with RAO pre- and post-challenge than in control horses (Figure 6B).

### *In vivo* NET formation in the airways of horses with RAO

**Figure 7:**
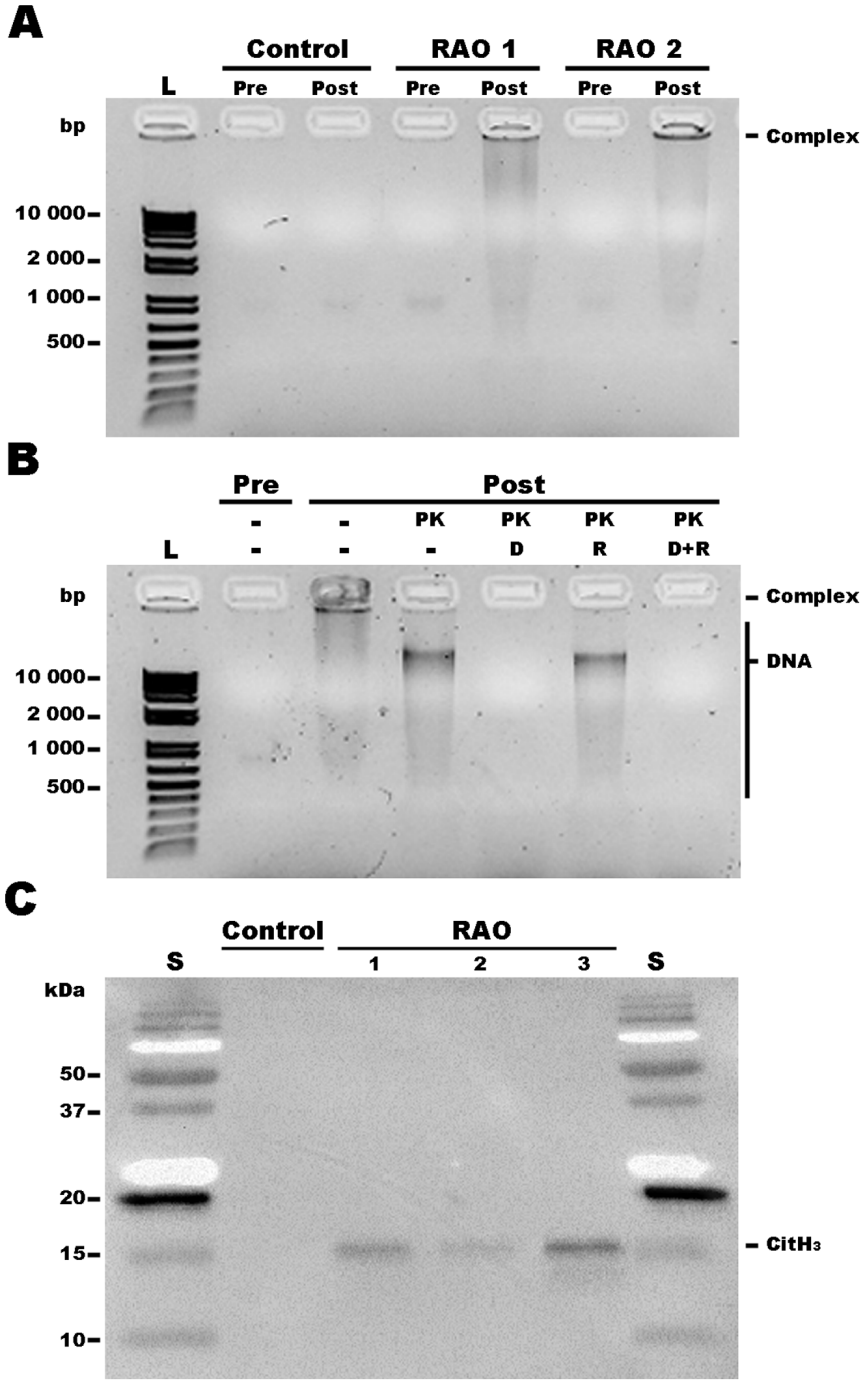
Characterization of NET-derived DNA-protein complexes in BAL fluid. (A) Nucleic acid-protein complexes were apparent as a high molecular weight band in agarose gels. The complexes were detected in animals with RAO post-challenge but not prior to exacerbation of RAO, and not in healthy control animals. (B) Proteinase K (PK) treatment released the proteins from the complexes and freed the nucleic acid, which migrate as lower molecular weight smear, confirming the presence of nucleic acid-protein complexes in the BAL. Addition of DNAse, but not (D) RNAse (R) abrogated the nucleic acid present in the BAL samples. Therefore, these samples contained DNA. (C) Western blot analysis revealed the presence of CitH_3_ (15 kDa) in samples that had HMW DNA-protein complexes. Collectively, these data indicate the presence of NETs in these BAL. L  =  1 Kb+ DNA ladder; S  =  Precision plus protein standard; bp  =  base pair; kDa  =  kilodaltons.

NET formation during neutrophilic inflammation in RAO has not been investigated. We determined whether there are NET-derived DNA-protein complexes in BAL fluid of horses without lung disease and with RAO using agarose gel electrophoresis. BAL fluid samples were collected from each horse pre- and post-exposure to dusty hay (which triggered exacerbated RAO in susceptible animals). After electrophoresis, pre- and post-challenge samples from control horses lacked high molecular weight nucleic acid (HMW) bands in BAL fluid, whereas such bands were present in samples from four of six animals with exacerbated RAO (Figure 7A). HMW bands in cell free preparations suggested the presence of nucleic acid-protein complexes. These complexes were detected only in samples from animals with exacerbated RAO (Table 1; Figure 7A). To evaluate the nature of these HMW bands, samples were treated with PK alone or in tandem with DNAse and/or RNAse. As shown in Figure 7B, incubation with PK alone detached the nucleic acid-protein complexes, highlighted by a shift in the molecular weight of the band. Disappearance of HMW bands following DNAse, but not RNAse, treatment confirmed that these complexes were made of DNA and proteins. Western blotting showed that the samples that had DNA-protein complexes also contained CitH_3_. Furthermore, the samples that lacked HMW DNA-protein complexes also lacked CitH_3_ (Figure 7C). These data are consistent with the interpretation that RAO BAL samples contained NETs. Notably, NETs were detected only in samples with high BAL neutrophil and total leukocyte concentrations, and low *SCGB1A1* and *SCGB1A1A* expression. These data suggest that during RAO, horse neutrophils release NETs into the airways.

**Table 1:** NETs, neutrophils and total leukocyte concentrations in BAL fluid samples from control and RAO horses.

	NETs		Neutrophils (10^3^/μL)		Total Leukocytes (10^3^/μL)	
	Pre	Post	Pre	Post	Pre	Post
Control						
Horse 1	-	-	8	90	415	822
Horse 2	-	-	3	160	321	1141
Horse 3	-	-	67	111	419	691
Horse 4	-	-	30	216	499	450
Horse 5	-	-	13	137	669	654
Horse 6	-	-	7	435	366	1358
Horse 7	-	-	9	118	423	538
Mean			20	181	445	808
SEM			8.55	44.98	42.8	125
						
RAO						
Horse 8	-	-	28	374	398	613
Horse 9	-	-	41	867	453	1294
Horse 10	-	+	18	773	434	1189
Horse 11	-	+	31	2218	510	2240
Horse 12	-	+	34	2078	428	3352
Horse 13	-	+	7	1998	726	2379
Mean			26	1385	492	1845
SEM			4.95	327.40	49.3	407

Presence of NETs was evaluated in BAL fluid from control and RAO-susceptible horses before (Pre) and after (Post) dusty hay challenge using agarose gel electrophoresis. BAL samples from control animal were negative (−) for the presence of protein-DNA complexes, while four of six samples from horses with exacerbated RAO (post) and most severe neutrophilic inflammation contained NETs (+).

## Discussion

SCGB 1A1 is the prototypic member of the secretoglobin family produced by specialized epithelial cells at the mucosal surface of the lungs and uterus. SCGB 1A1 has anti-inflammatory and immunomodulatory properties due to inactivation of PLA2, sequestration of pro-inflammatory cytokines and interference with leukocyte chemotaxis [7], [30], [31]. Inhibition of PLA2 by SCGB 1A1 limits generation of neutrophil activating arachidonic acid metabolites, and is considered a mechanism of reducing inflammation and limiting neutrophil-induced lung injury in acute respiratory distress syndrome [32]. In addition to indirect effects on neutrophils, direct interaction was suggested by detecting SCGB 1A1 in lung neutrophils of animals with neutrophilic inflammation by immunoelectron microscopy [27]. SCGB 1A1 is depleted in bronchial epithelium and airway fluid of human asthmatics [33], [34] and horses with stable RAO, an asthma-like condition characterized by intense neutrophilic inflammation of the airways [17]. It was recently identified that *SCGB1A1* genes are triplicated in the equid family, and that two of the genes are differentially expressed and give rise to distinct proteins [18], [19]. SCGB 1A1 and 1A1A differ by 13 of 70 amino acids, with 7 of the13 substitutions in the region that forms the hydrophobic pocket [19]. The binding pocket of SCGB 1A1A is smaller than that of SCGB 1A1, and substitutions include amino acids with different biochemical properties. Theoretical isoelectric points (pI) of SCGB 1A1 and A1A1 are 4.90 and 6.48, further suggesting potentially different biological functions. Hence, we here sought to determine whether the different SCGB proteins have unique effects on functional properties of neutrophils and NET formation in a naturally occurring model of inflammatory lung disease.

Incubation of neutrophils with recombinant SCGB 1A1A but not SCGB 1A1 enhanced ROS production and increased phagocytosis. Receptors whereby SCGB 1A1 directly interacts with cells to affect function have not clearly been identified. It was reported that SCGB 1A1 enters renal tubular cells via binding to cubilin, a large peripheral membrane protein, and that subsequently this complex interacts with the endocytic lipocalin receptor megalin [35]. Expression of these receptors by non-epithelial cells has not been identified; hence interaction of SCGB with other molecules on neutrophils appears more likely. The structurally related SCGB 3A2 binds to macrophage scavenger receptor with collagenous structure (MARCO) present on alveolar macrophages, and competes with LPS for binding to MARCO and bacteria [36]. Similar innate pattern recognition receptors are plausible candidate SCGB 1A1 receptors. SCGB 1A1 had been reported to inhibit neutrophil oxidative burst, but studies preceded identification of multiple expressed *SCGB1A1* genes in horses [27]. The *in vivo* significance of functional effects of either protein will further depend on concentration and local conditions such as pH, but *in vitro* assays suggest specific properties that functionally distinguish between the isoforms. Future studies will need to determine *in situ* concentration of each protein during active disease.

Phagocytosis was significantly increased by incubation with 250 ng/mL of SCGB 1A1A but not SCGB 1A1. Increased neutrophil phagocytosis of opsonized 1 µm beads suggests that SCGB 1A1A may interact with phagocytic receptors, immunoglobulins or complement products such as C3bi. Intravenous administration of SCGB 1A1 reduced complement deposition and glomerulonephritis in a mouse model, and mice lacking SCGB 1A1 developed IgA nephropathy, suggesting that SCGBs *in vivo* regulate function of these immune mediators [37], [38]. Phagocytosis of inert particulate does not necessarily initiate a subsequent pro-inflammatory neutrophil response, and may rather reflect removal of apoptotic cells and cell debris [39]. Since *in vitro* concentrations of SCGB corresponded to estimates of physiologic levels in airway secretions, effects on oxidative burst and phagocytosis should also be further investigated *in vivo* (38, 39).

Numerous reports have identified effects of SCGB 1A1 on cell migration and chemotaxis of leukocytes, fibroblasts, smooth muscle and neoplastic cells [26], [31], [40]–[43]. In agreement with these observations, our findings showed both SCGB 1A1 and 1A1A strongly reduced IL-8-stimulated chemotaxis of blood-derived neutrophils in a dose-dependent manner. SCGB 1A1A was a more potent inhibitor of cell migration. A postulated mechanism whereby SCGB 1A1 affects chemotaxis is binding to the formyl-met-leu-phe (fMLP) receptor FPR2, which blocks association with bacterial-derived fMLP. SCGBs may also interfere with binding of IL-8 to its CXCR1 or CXCR2 receptor. Since despite much effort receptors for SCGB 1A1 have not been conclusively identified and the mechanisms for chemotaxis inhibition remain elusive. Hall and colleagues [44] reported that BAL fluid from RAO-affected animals enhanced chemotaxis. However, BAL fluid contains many mediators produced by bronchial epithelium or leaked into the airways from vasculature, and such assay does not allow dissection of individual protein effects. SCGBs are highly abundant in secretions of the lower bronchi and bronchioles, and animals with RAO have overall reduced club cells and SCGB production [17], which may facilitate neutrophil chemotaxis to these sites of most intense inflammation. Hence, SCGB-mediated reduction of neutrophil influx is likely a protective anti-inflammatory property of these proteins.

SCGB pre-incubation of neutrophils activated by calcium flux significantly reduced *ex vivo* NETosis. NETs trap microbes or particulate matter, and in turn enhance their phagocytosis and elimination, but constituents of NETs such as citrullinated histones and extracellular MPO are also immunogenic and cytotoxic [45]. NETs in the equine male reproductive tract were associated with neutrophil activation and impaired fertility [46], and excessive NETs may have deleterious effects in the lung [25]. SCGBs likely contribute to balancing NETosis in the lung, a function to be further explored.

NETs have previously been identified in the asthmatic airways of humans but not horses [47]. In humans, both neutrophilic and eosinophilic extracellular traps occur [46], but neutrophilic inflammation characterizes types of human asthma less responsive to therapy, which is similar to equine RAO. Measurement of *SCGB1A1* and *SCGB1A1A* transcripts in bronchial biopsies, and total SCGB in BAL fluid, showed that SCGBs were decreased in animals with RAO, and in particular transcripts of *SCGB1A1* were barely detectable in RAO horses. SCGB 1A1 may be more important for modulating neutrophil responses in the airway, but depletion in chronic inflammation abrogates *in vivo* effects. We identified NETs in BAL samples of horses with acutely exacerbated RAO and severe neutrophilic inflammation, but not in the same horses during disease remission or in control horses exposed to the same stimulus. The *in vivo* stimuli or significance of NETs are incompletely defined, but a wide range of microbial components and inflammatory mediators such as GM-CSF, ROS, IL-8 and MIP-2 may induce NETosis [25]. Since agents that incite asthmatic responses in horses include LPS and fungal spores [48], and the bronchial epithelium responds with abundant IL-8 production [49], it is not surprising that neutrophils are activated *in vivo* and form NETs. From data presented here it appears that NETs are present in the most severe the inflammatory states, but the precise role of NETs in RAO remains to be determined.

RAO is a severe neutrophilic lung inflammatory disease induced by air containing dust, fungal spores and bacterial components. Bronchial epithelial club cells and their SCGB products are markedly reduced in affected animals. We here show that SCGB 1A1 and 1A1A have differential effects on neutrophils, and that duplication and evolution of the *SCGB1A1* gene may have resulted in differential effects on neutrophil responses. A global understanding of the molecular targets and pathways affected by SCGB proteins may help development of pharmacological interventions to counteract pathogenic neutrophilic inflammation, and will be the subject of future studies.
